# Exosomal lncRNA Mir100hg derived from cancer stem cells enhance glycolysis and promote metastasis of lung adenocarcinoma through mircroRNA-15a-5p/31-5p

**DOI:** 10.1186/s12964-023-01281-3

**Published:** 2023-09-21

**Authors:** Lei Shi, Bowen Li, Yuhan Zhang, Yuting Chen, Jiyu Tan, Yan Chen, Jie Li, Meng Xiang, H. Rosie Xing, Jianyu Wang

**Affiliations:** 1 State Key Laboratory of Ultrasound in Medicine and Engineering, College of Biomedical Engineering, Chongqing Medical University, Chongqing, 400016 China; 2 Chongqing Key Laboratory of Biomedical Engineering, Chongqing Medical University, Chongqing, 400016 China; 3 Institute of Life Sciences, Chongqing Medical University, Chongqing, 400016, China

**Keywords:** Exosomal lncRNA, Mir100hg, miR-15a-5p, miR-31-5p, Glycolysis

## Abstract

**Background:**

Exosomes are a new class of molecular entities in the metastatic microenvironment, which can mediate bidirectional communication between cells. While exosomes-mediated interactions between tumor cells and other cell populations in the tumor microenvironment have attracted most attention, little is known about the significance of exosomes in mediating the interaction between non-stemness cancer cells and cancer stem cells during cancer progression.

**Methods:**

The structure, sequence and downstream target miRNAs of lncRNA Mir100hg were predicted by online web resources. The bioinformatics prediction results were validated with experimental verification: exosome tracing, electron microscopy, Luciferase assay, metabolomics sequencing and mouse tail vein model of pulmonary metastasis. A complex regulatory network of "cancer stem cells-exosomal lncRNA-non-stem cancer cells" was constructed.

**Results:**

This study demonstrates firstly that lncRNA Mir100hg is upregulated in lung cancer stem cell LLC-SD (Lung cancer stem cells) and can be delivered to non-stemness cancer cells LLC (Lewis lung cancer cells) via exosomes. In LLC, Mir100hg targets miR-15a-5p and miR-31-5p which leads to the increase of the global glycolytic activity of lung cancer cells and consequently, the enhancement of their metastatic capability.

**Conclusion:**

We delineated a complex regulatory network that utilized by cancer stem cells to transfer their high metastatic activity to the low-metastatic non-stemness cancer cells through exosomal Mir100hg, thereby providing new mechanistic insights into the communication between two heterogeneous tumor cells.

Video Abstract

**Supplementary Information:**

The online version contains supplementary material available at 10.1186/s12964-023-01281-3.

## Background

Lung cancer is the leading cause of death among all malignancies, which is closely related to its propensity for metastasis [1]. Cancer stem cells (CSCs) are deemed as the fundamental "seeds" for tumor development, progression, and recurrence [2]. CSCs possess self-renewal capacity, divergent differentiation, high tumorigenicity, and therapy resistance, which contribute to the evolution of heterogeneous tumor components [3]. Tumor heterogeneity manifests through genetic and molecular changes that result in varying tumor growth rates, invasion and metastasis potential, drug and radiotherapy sensitivity, and prognosis [4]. Recently, the interactions between immune cell populations and tumor cells infiltrating the microenvironment have garnered attention [5, 6]. However, the exchange of exosomes between non-stemness cancer cells and CSCs and its significance in cancer progression are poorly investigated.

In our previous studies, we have shown that melanoma stem cells with high metastatic potential can transmit multiple cancer-promoting miRNAs to the non-stemness cancer cells through exosomes, significantly enhancing their metastatic colonization capability [7, 8]. Similarly, tumor cells at the primary site can regulate the surrounding microenvironment through exosomal lncRNAs, promoting the dissemination to distant sites and escaping immune survilliance [9, 10]. Furthermore, molecular data related to extracellular vesicles (EVs) containing RNA molecules have surged with the discovery of deep sequencing technology. Several databases have been made accessible to the scientific community, enabling bioinformatics analysis for comprehending the roles of lncRNAs in EVs and intercellular communication [11, 12]. In the present study, we constructed a complex regulatory network of "Cancer stem cells- Exosomal lncRNA—Non-stemness cancer cells" using web resources to predict the structure, sequence, and downstream target miRNAs of lncRNA. The bioinformatics prediction results were combined with experimental verification to delineate the exosome-mediated intercellular communication and intracellular regulatory circuits between two heterogeneous tumor cell populations, gaining new insights into exosome communication and transmission between non-stemness cancer cells and CSCs.

Mir100hg is highly expressed in multiple tumors and is closely associated with biologic behaviors, including proliferation, cell cycle, migration and invasion, metastasis, drug resistance, and EMT [13–16]. It has been reported that Mir100hg can participate in tumorigenesis as a promoter of RNA-binding proteins, a structural component supporting the formation of nucleic acid and protein complexes, and a precursor of miRNAs [17–20]. These mechanisms are involved in the regulation of a variety of biological processes through WNT, MAPK, TGF-β, HIPPO signaling pathways [16, 18, 21, 22]. However, little is known about the role of Mir100hg in lung cancer, and the relationship between Mir100hg and tumor metabolism and tumor-associated exosomes has not been reported.

Here, we combine bioinformatics analysis with experimental validation to demonstrate firstly that lncRNA Mir100hg is upregulated in lung cancer stem cell LLC-SD and can be delivered to non-stemness cancer cells LLC via exosomes. In LLC, Mir100hg targets miR-15a-5p and miR-31-5p which leads to the increase of the global glycolytic activity of lung cancer cells and consequently, the enhancement of their metastatic capability. We have delineated a complex regulatory network that utilized by cancer stem cells to transfer their high metastatic activity to non-stemness cancer cells through exosomal Mir100hg, thereby providing new mechanistic insights into the communication between two heterogeneous tumor cells. Although no substantial progress has been made in the clinical application of Mir100hg, exosomal lncRNA is stable in body fluids, and Mir100hg could potentially serve as a tumor diagnostic and prognostic marker.

## Methods

### Cell culture

LLC and HEK-293 T cells were cultured in Dulbecco's Modified Eagle Medium (Gibco) supplemented with 10% Fetal bovine serum (Gibco). LLC-SD was cultured in DMEM/F12 medium (Gibco) supplemented with 1%B27 (Gibco).LLC-SD cells were generated as shown in previous studies [23]. All cells were cultured at 37° C in 5% carbon dioxide.

### Extraction of exosomes

Exosomes were extracted by differential centrifugation. The cell supernatant was collected and centrifuged at 4° C sequentially with different speeds of 800 × g/5min, 2000 × g/10min, 9000 × g/50min, 100,000 × g/90min. The precipitate were resuspended in PBS at 100,000 × g/90min. The exosomes were suspended in PBS and filtered through a 0.22μm filter membrane to remove impurities. The collected exosomes were subjected to subsequent authentication and functional experiments.

### Western blot

Cells of 1 × 10^6^ to 1 × 10^7^ were collected and added to 100 to 300ul of RIPA lysate (Solarbio) and mixed until there was no obvious solid. The mixture was vortexed for 1min and then placed on ice for 10min, during which vortexing was repeated every 3min to thoroughly lyse the cells. The lysis mixture was then placed in a centrifuge and centrifuged at 11,000 × g. The upper clear liquid was aspirated into a new eppendorf tube. Sample concentrations were leveled using a BCA Kit (CWBIO). Add loading buffer (5 × SDS) according to the amount of liquid, and boil. 30 to 50 μg protein were loaded in each loading well. The two-step voltage was set to 80V-30min/120V-1h, and the PVDF membrane was used for trarsmembrane at the end of electrophoresis, and the voltage was set to 100V-1h. Blocking solution was configured with 5%BSA, and visualization shots were taken at the end of antibody incubation.

### RT-PCR

Total intracellular RNA(Yeasen), exosomal RNA(Qiagen), and nucleoplasmic isolate RNA(NORGEN) were extracted using kits. Reverse transcription was performed using Prime Master Mix (Vazyme). The RT-qPCR reaction was performed using universal SYBR (Vazyme).Detailed primer sequences are listed in supplementary data.

### Cell migration and invasion assays

Matrigel gel (BD Biosciences) was stored in a 4° C refrigerator overnight for thawing. The gel was diluted to 1 ml with serum-free cell medium before use, and 100 μl of the liquid was uniformly dropped onto the upper surface of the transwell chamber PET membrane. The transwell(Corning) were placed at 37° C for approximately 1 h. Cells were resuspended in 300ul of DMEM medium to be added above the chamber, and 800ul DMEM (20% Fetal bovine serum (FBS)) was added below the chamber (bottom of 24-well plate). After 20 h of incubation, the Transwell chambers were removed. The lower surface of the Transwell chamber was immersed in 70% methanol solution and fixed for 30 min before staining with crystal violet; after staining, excess crystal violet was washed with PBS and observed under a microscope. Five central and five peripheral visual fields were photographed and statistically analyzed.

### Fluorescence in situ hybridization(FISH)

Using the principle of base complementary pairing of nucleic acid molecules, the artificially designed exogenous nucleic acid fragment (Genepharma, Red) was complementary paired with Mir100hg in cells. The nucleus was labelled with DAPI (Beyotime, Blue). RNA localization was observed under the fluorescence microscope (ANDOR, Dragonfly200).

### Exosome tracing

Exosomes were labeled using PKH26 (Warbio, red). After the extraction, exosomes were resuspended in 100ul PKH26 diluted in PBS and stained at room temperature for 5min. Fluorescently labeled exosomes were obtained by washing twice with PBS and ultracentrifuging (100,000 × g/90min). PKH26-labeled exosomes were co-cultured with the recipient cells at 37°C for 6h. Localization of exosomes within the recipient cells was observed under the confocal microscope (ANDOR, Dragonfly200).

### Co-culture experiments

LLC cells were seeded at a density of 70% for the experiment. LLC cells were washed twice with 1XPBS and replaced with fresh medium before co-culture. Next, transwell co-culture chambers with a 0.4um pore size (Corning) were spread above a 6-well plate. LLC-SD cells (1*10^4) were added to the trasnwell chambers and resuspended in F12/B27 medium. After co-culture for 24 h, the upper transwell chambers were removed, and the lower LLC cells were digested with trypsin and used for migration and invasion assay.

### RNA-binding protein immunoprecipitation (RIP)

A number of 1*10^7 cells per RNA immunoprecipitation sample was prepared by adding 1 ml of lysis buffer to the culture dish and scraping the cells into a 1.5 ml EP tube using a cell scraper. Next, the cells were lysed on ice for 10 min and vortexed for 10 s every 3 min. After lysis, the cells were centrifuged at 13000rmp for 10 min in a cryogenic centrifuge at 4°. Clear supernatant fluid from the tubes was aspirated and divided into Input (100ul) and IP (800ul) groups. The supernatant of the Input group was immediately placed in the -80° refrigerator and used the next day. The IP group was divided into AGO2 group and IgG group with 400ul each. The magnetic beads incubated with the target antibody (AGO2/IgG) were added to the beads and rotated overnight for binding. The next day, the magnetic beads were retained to discard the supernatant, and after washing the magnetic beads for 5–10 passes, the lysate was added. PureBinding®RNA Immunoprecipitation Kit (GENESEED) was used for all the reagents and columns.

### Animal experiments

Nod-SCID mice were injected with 1 × 10^6^ cells via the tail vein. Animals were randomly assigned to each group, and mice were sacrificed and autopsied 5 weeks later.

Exosome in vivo experiments: Three groups were set up, namely PBS, sh-NC-exo and sh-Mir100hg-exo. LLC-GFP cells labeled with green fluorescence (1 × 10^6^) were injected into mice via tail vein on day 0. Then, the same volume of PBS and PBS diluted exosomes derived from LLC-SD-sh-NC and LLC-SD-sh-Mir100hg cells were injected on days 7, 13, 15, 19, and 21, respectively, for a total of 5 injections of exosomes at a concentration of 1*10^9 / particle each time. The mice were sacrificed on day 28, and the tumor in the lung and other areas was observed by fluorescent flashlight.

Pulmonary metastases were observed using Sellstromz87(LUYOR, 3430-RB, Shanghai, China).

### Dual luciferase assay

Plasmids of lncRNA Mir100hg were obtained from Hanbio. HEK-293 T cells were used for the experiments. Luciferase system was prepared as follow: Solution A: 10 μl(DMEM) + 0.8 μl(plasmid) + 0.25 μl(mimics/NC); Solution B: 10 μl (DMEM) + 0.24 μl (lip2000). The fluorescence value was detected by VARIOSKANLUX (Thermo).

### miRNA target gene prediction

Annolc(http://annolnc.cbi.pku.edu.cn) and miRDB website (https://www.mirdb.org) were used to predict the miRNAs that are subjected to Mir100hg targeting. TargetScan (https://www.tar getscan.org) and miRDB were used to predict the possible target genes of miRNA. Using Sangerbox (http://sangerbox.com/home.html) to perform the GO and KEGG (Kyoto Encyclopedia of Genes and Genomes).

### Metabonomics and mass spectrometric analysis

OE-Mir100hg-LLC and OE-Vecter-LLC cells were collected for targeted energy metabolite sequencing. OE-miR-15a-5p/31-5p-OE-Mir100hg-LLC and OE-miR-15a-5p/31-5p-OE-Mir100hg-LLC cells were collected for non-targeted metabolomics sequencing, and the detection and analysis process were completed at the Mass Spectrometry Center of Laboratory of Maternal–Fetal medicine of Chongqing Medical University.

### Data resource description and processing

The LUAD clinical dataset was downloaded from the TCGA. GSE66616 was obtained from GEO (Gene Expression Omnibus). It contains 66 samples, including cocultures of invasive or non-invasive NSCLC cell lines and various types of fibroblasts. To identify changes in gene expression in the sequencing data, we used the limma package in R (4.2.2) to identify differentially expressed genes.

### Statistics

Migration and invasion counts were performed using Image J, and outcome statistics were quantified using mean ± standard deviation (mean ± SEM). T test was used to compare the differences between the two groups, and ANOVA was used to compare the differences between multiple components. Statistical graphs were plotted using GraphPad Prism6.0 (ns, not significant; *, *P* < 0.05; **, *P* < 0.01; ***, *P* < 0.001).

## Results

### Characterization of exosomes derived from LLC and LLC-SD

**Fig. 1:**
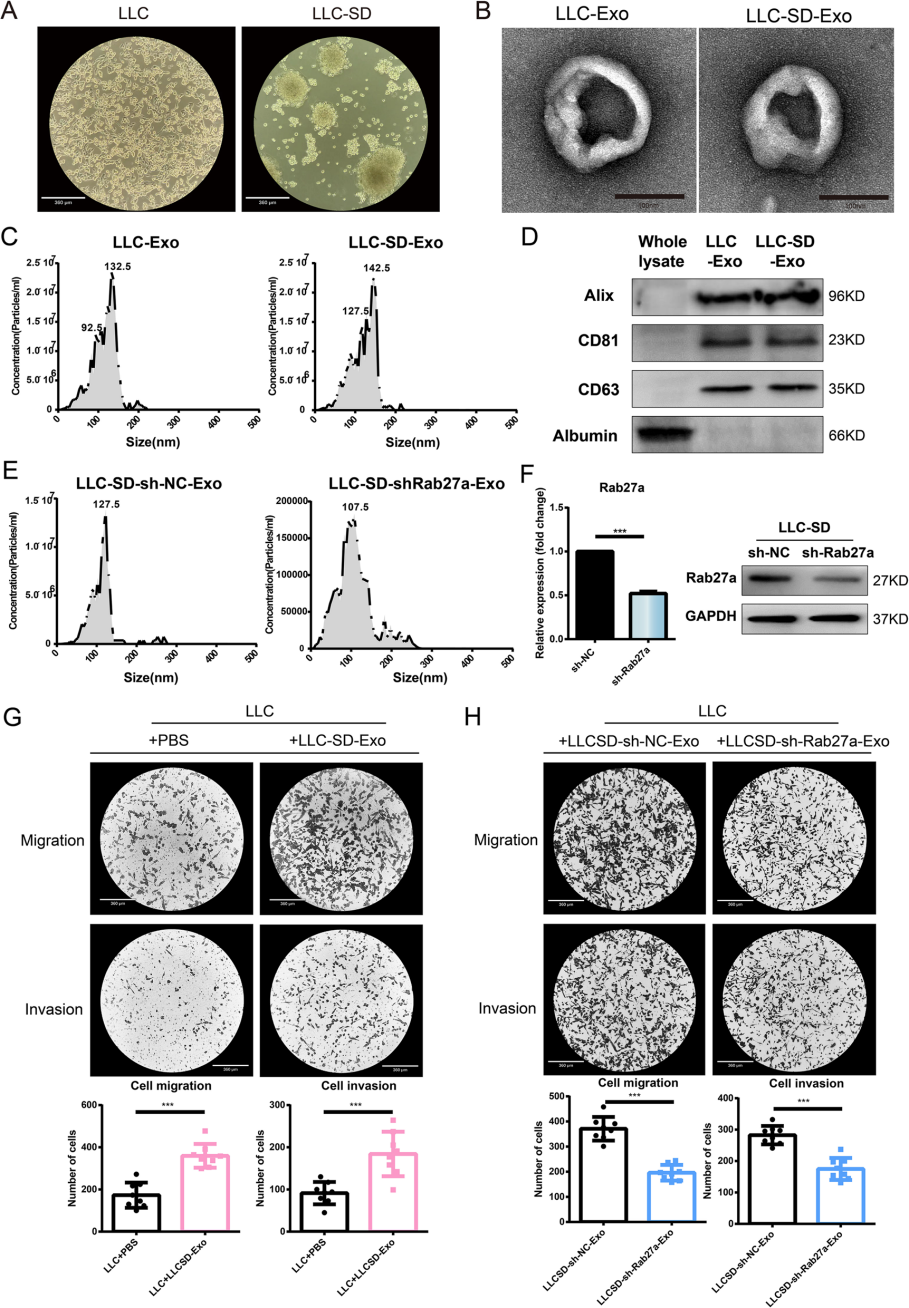
Exosomes derived from LLC-SD enhanced the metastatic ability of non-stemness LLC cells in vitro.** A** The cell morphology of LLC and LLC-SD was observed under light microscope; **B** Exosomes isolated from LLC and LLC-SD were observed by transmission electron microscope; **C** The particle size and concentration of exosomes were determined by Nanoparticle Tracking Analysis. **D** Exosome marker proteins were detected by western blot. **E**–**F** Efficient Rab27a knockdown at mRNA and protein levels; **G** LLC-SD-exosomes enhanced the migration and invasion ability of LLC; **H** Effects of shRab27-LLC-SD-Exosomes (50μg,24 h) on the migration and invasion of LLC. (*p < 0.05, **p < 0.01, ***p < 0.001)

In a previous study, we isolated lung cancer stem cells (LLC-SD) from the Lewis lung cancer cells (LLC) by an eight-round sphere-forming assay and demonstrated that LLC-SD possess enhanced stemness and tumorigenic ability both in vitro and in vivo [23]. LLC were spindle-shaped and grew adherent, while LLC-SD cells were spherical and grew in suspension (Fig. 1A). Next, the exosomes secreted by LLC and LLC-SD were extracted by differential centrifugation, and the morphology of the exosomes was observed by transmission electron microscopy. The exosomes showed a characteristic double-concave disc shape (Fig. 1B). The diameter range of the extracted exosomes was mainly concentrated between 40 and 150nm by Nanoparticle Tracking Analysis (Fig. 1C). The purity of the exosomal preparations was confirmed by the expression of the classical exosome markers (Alix, CD81, CD63) via Western Blot analysis (Fig. 1D).

### Exosomes derived from LLC-SD augments the metastatic ability of non-stemness cancer cells in vitro

Studies have shown that intratumoral cellular heterogeneity contributes to tumor metastasis, treatment resistance and affects patient survival [24, 25]. CSCs are part of tumor heterogeneity. However, whether lung CSCs can affect the metastatic ability of non-CSC lung cancer cells through CSC-secreted exosomes remains to be explored and was the focus of the present study. We investigated the effect of exosomes secreted by LLC-SD on the migration and invasion of LLC cells. We found that adding exosomes secreted by LLC-SD cells (LLC-SD-Exo) to LLC cells in the co-culture significantly enhanced the migration and invasion ability of LLC cells in vitro (Fig. 1G). Rab27a regulates the secretion of exosomes in A549 cells [26, 27]. The specificity of LLC-SD-exo for the enhanced migration and invasion ability of co-cultured LLCs was confirmed by stable knock down of Rab27a in LLC-SD by shRNA (Fig. 1E-H). Therefore, we hypothesized that LLC-SD-Exo could promote the migration and invasion ability of LLC cells by exosomal delivering of certain encapsulated functional contents.

### Exosomal lncRNA Mir100hg secreted by LLC-SD enhances the metastatic ability of LLC

**Fig. 2:**
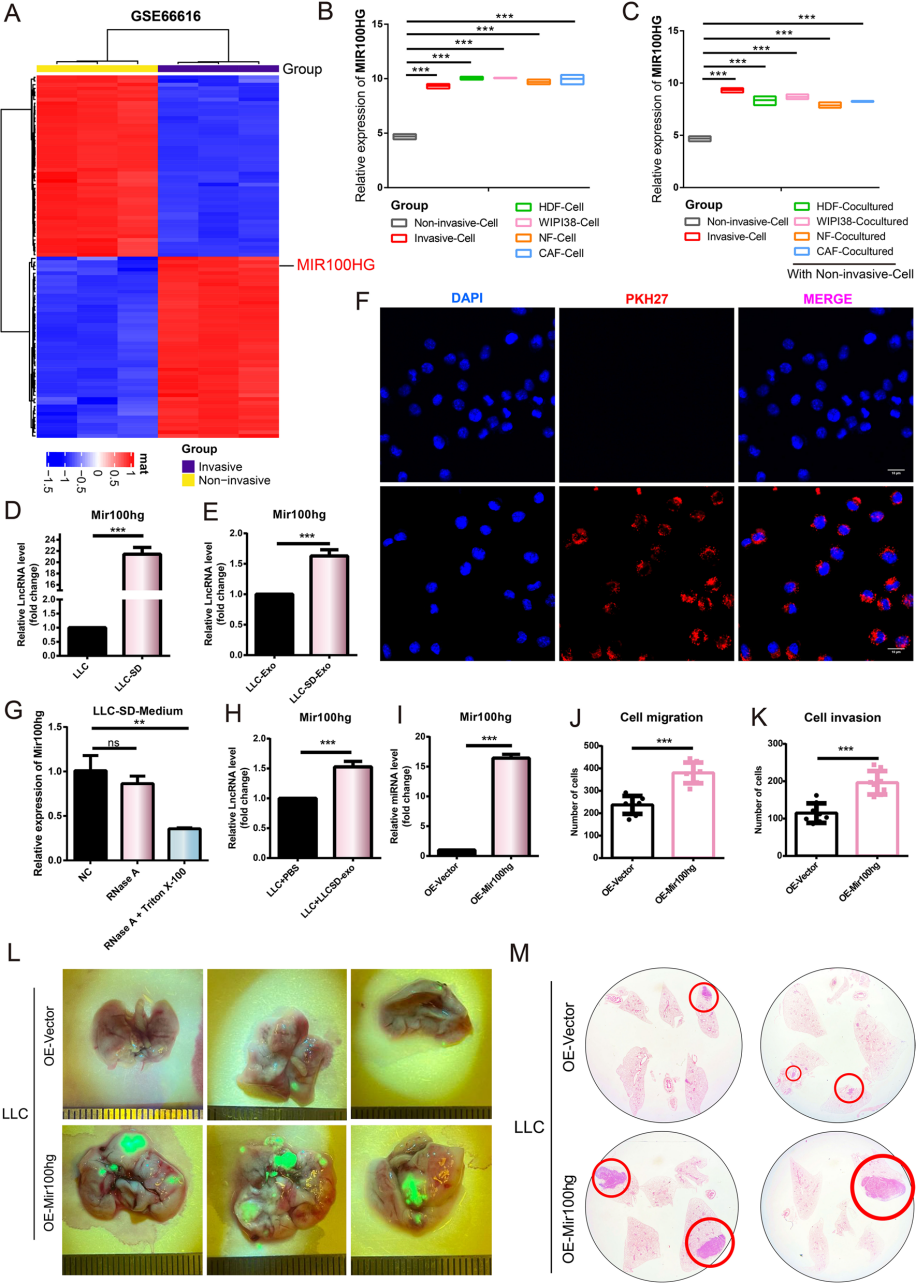
lncRNA Mir100hg promotes the metastatic activity of lung cancer cells in vitro and in vivo. **A** Heatmap of differentially expressed genes in GSE66616 dataset, MIR100HG(Red); **B** Boxplot of MIR100HG expression differences between non-invasive lung cancer cells and invasive lung cancer cells and fibroblasts; **C** Boxplot of MIR100HG expression levels in non-invasive lung cancer cells after co-culture with different fibroblast cells; **D** Differences in Mir100hg expression between LLC-SD and LLC cells; **E** Differences in Mir100hg expression between the exosomes of LLC-SD and LLC’s; **F** LLC-SD-exosomes (labeled with PKH26, Red) could be delivered to LLC cells; **G** The expression of Mir100hg in the supernatant treated with RNase A (2mg/mL) alone or in combination with Triton X-100 (0.1%) for 20 min was analyzed by RT-PCR. **H** Expression of Mir100hg in LLC after co-incubation of LLC-SD-exosomes for 20h; **I** The efficiency of Mir100hg overexpression in LLC; **J**-**K** Column chart of migration and invasion cells; **L** Lung metastasis was observed after tail vein injection of OE-Mir100hg-LLC (green). **M** HE staining was used to observe and quantify lung metastases. (*p < 0.05, **p < 0.01, ***p < 0.001)

Using bioinformatics analysis of GSE66616, a dataset of non-small cell lung cancer, we found that the expression of lncRNA MIR100HG was elevated in the invasive cells (Fig. 2A). We extracted the relative expression of MIR100HG in lung cancer cells and fibroblasts from the GSE66616 dataset, and compared the expression of MIR100HG in non-invasive and invasive lung cancer cells with other fibroblasts (Fig. 2B). The results showed that the expression of MIR100HG was relatively low in non-invasive lung cancer cells, while it was highly expressed in invasive lung cancer cells and other fibroblasts. We then compared the expression of MIR100HG in non-invasive lung cancer cells after co-culture with other fibroblasts. Interestingly, MIR100HG expression of non-invasive cells increased to a level similar to that of invasive cells after co-culture with various types of fibroblasts (Fig. 2C). This result implies that MIR100HG may somehow be transmitted between the two types of cells and lead to corresponding changes in its intracellular expression. We first examined the expression of Mir100hg in lung cancer stem cells (LLC-SD) versus non-stemness lung cancer cells (LLC), and we found that Mir100hg was overexpressed in LLC-SD compared to that in LLC cells (Fig. 2D). Similarly, the expression of Mir100hg in the exosomes secreted by LLC-SD was also higher than that of exosomes secreted by LLCS (Fig. 2E). Next, we performed exosome tracing experiments to determine that exosomes secreted by LLC-SD could be taken up by the LLC-cells (Fig. 2F). When Triton X-100 and RNase were added simultaneously to the cell supernatant to disrupt membranous compartments, we found that the expression of Mir100hg was significantly reduced, indicating that Mir100hg was present in the cell supernatant and protected by membranous exosomes (Fig. 2G). When exosomes from LLC-SD were added to the co-culture of LLC cells, the expression of Mir100hg in LLC cells also increased (Fig. 2H). These results indicate that Mir100hg is overexpressed in LLC-SD and can be delivered to LLC cells through exosomes, resulting in the increased expression of Mir100hg in LLC cells. We firstly determined the oncogenic role of Mir100hg in LLC cells, after overexpression of Mir100hg in LLC cells (Fig. 2I), the migration and invasion ability of LLC cells was enhanced (Fig. 2J-K, Supplementary Fig. 1). In addition, we established a Nod-Scid mouse model of pulmonary metastasis via the tail vein injection of tumor cells (Materials Methods—Animal Experiments). More macroscopic metastases and larger metastatic areas were observed in the OE-Mir100hg group (Fig. 2L). The results of lung HE staining showed that the OE-Mir100hg group had more extensive lung metastasis (Fig. 2M).

**Fig. 3:**
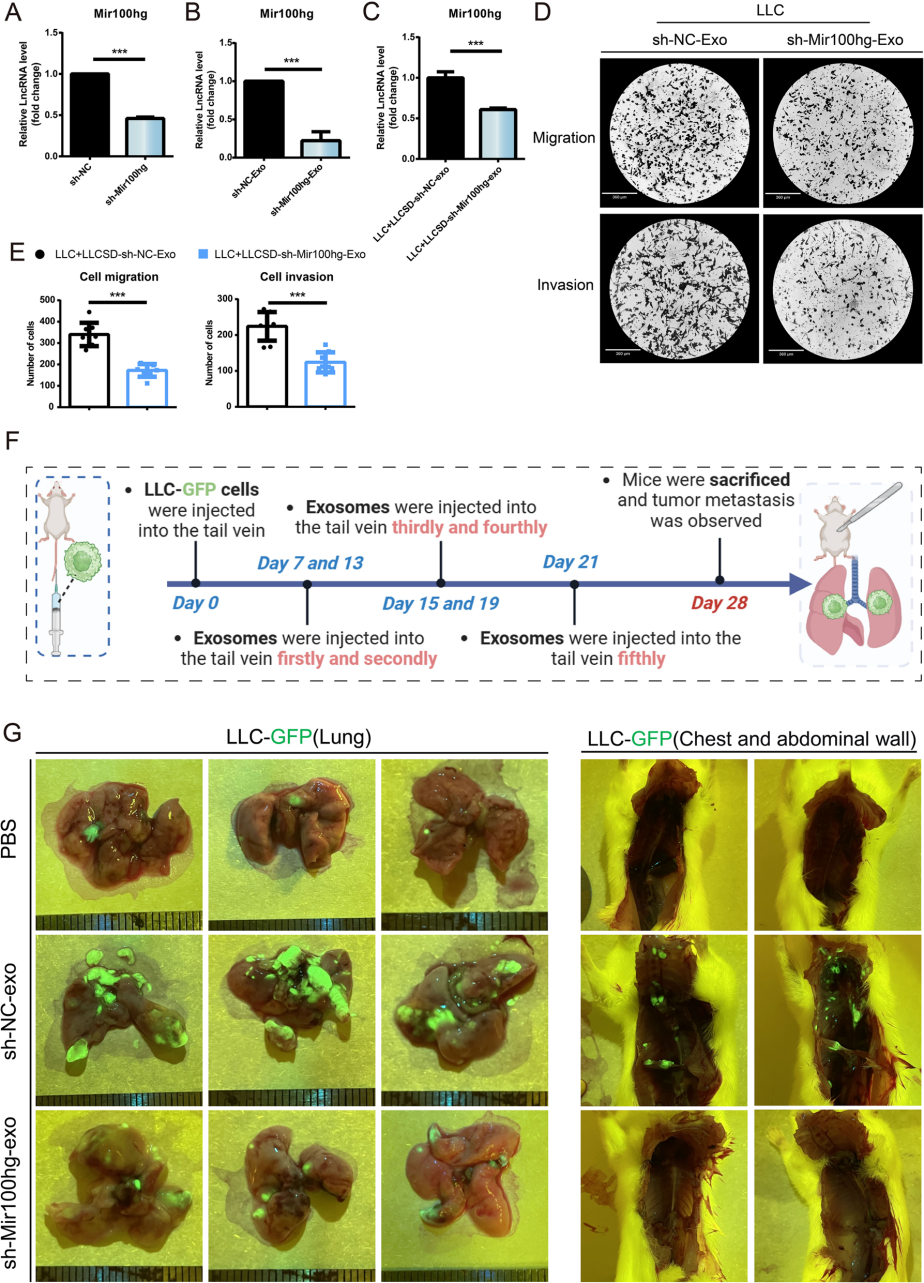
Exosomal lncRNA Mir100hg secreted by LLC-SD enhanced the metastatic ability of LLC. **A** The efficiency of Mir100hg Knockdown in LLC-SD; **B** Expression of Mir100hg in LLC-SD-shMir100hg-Exosomes; **C** Expression of Mir100hg in LLC after co-cultured with LLC-SD-shMir100HG-exosomes for 20h; **D** The migration and invasion ability of LLC after co-culturing with LLC-SD-shMir100hg-exosomes for 20 h; **E** Column chart of migration and invasion cells; **F** Schematic representation of exosome animal experiments; **G** Tumor metastasis in the lung and chest and abdominal wall was observed in mice after tail vein injection of different cell-derived exosomes (green). (*p < 0.05, **p < 0.01, ***p < 0.001)

To determine the effects of delivery of Mir100hg by exosomes from LLC-SD to LLC-cells, we firstly knocked down Mir100hg in LLC-SD (Fig. 3 A) and observed a corresponding decrease of the expression of Mir100hg in exosomes of LLC-SD (Fig. 3B). More importantly, the expression of Mir100hg in LLC was reduced compared with the control group after co-culturing with the LLC-SD exosomes knocking down Mir100hg (Fig. 3C), accompanied by weakened migration and invasion ability (Fig. 3D-E). We then evaluated the effect of Mir100hg in LLC-SD derived exosomes on tumor metastasis in mice (Fig. 3F-G). The lung tumors of the PBS-treated mice grew in a single focus, and no tumor metastasis was observed in the chest and abdominal wall. To our surprise, the sh-NC-exo group showed a trend of multifocal tumor growth and extensive tumor metastasis in the chest and abdominal wall, and tumor metastasis was found in the heart, spleen capsule, liver and kidney of this group of mice (Supplementary Fig. S3). This is consistent with the results of transwell migration and invasion assay of lung cancer cells treated with LLC-SD-exo in our manuscript, which showed that treatment of lung cancer stem cell exosomes could significantly enhance the metastatic ability of lung cancer cells (Fig. 1). However, although the lung tumors in the sh-Mir100hg-exo group showed multifocal growth distribution, the size and number of tumors were far less than those in the sh-NC-exo group. Collectively, these results indicate that delivery of Mir100hg by exosomes from LLC-SD to LLC cells can enhance the metastatic colonization efficiency of LLC cells both in vitro and in vivo.

### Mir100hg augments metastatic activity of lung cancer cells by targeting miR-15a-5p/miR-31-5p

**Fig. 4:**
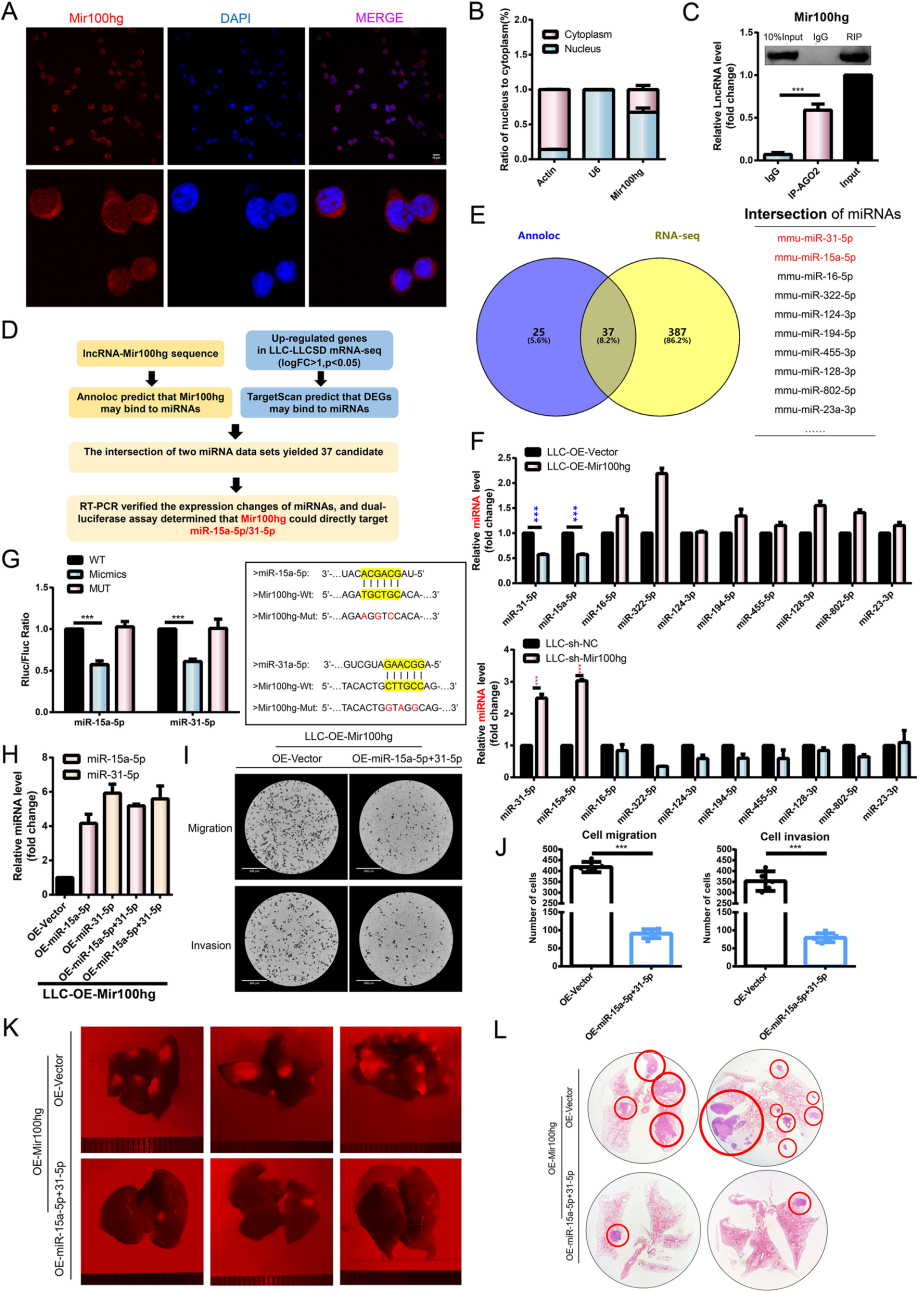
Mir100hg augmented lung cancer metastasis by targeting miR-15a-5p/miR-31-5p. **A** Mir100hg was localized in both the nucleus and cytoplasm as determined by FISH (Mir100hg, Red); **B** The localization of Mir100hg in both nucleus and cytoplasm was confirmed by RNA separation assay(Actin was used as a cytoplasmic control and U6 as a nuclear control; **C** Direct binding of Mir100hg to AGO2 was determined by RIP; **D** Schematic representation of Mir100hg targeting miR-15a-5p and miR-31-5p inferred from Mir100hg sequence and sequencing data from LLC-SD and LLC; **E** 37 candidate miRNAs screened from Annolc and sequencing data **F** The expression of miRNAs was examined by RT-PCR in OE-Mir100hg-LLC and sh-Mir100hg-LLC cells; **G** Luciferase assay confirmed that Mir100hg could targeted miR-15a-5p and miR-31-5p directly; **H** The efficiency of overexpression of miR-15a-5p and miR-31-5p and co-overexpression of the two miRNAs in OE-Mir100hg-LLC cells; **I**-**J** Migration and invasion of co-overexpression of miR-15a-5p and miR-31-5p in OE-Mir100hg-LLC cells; **K** Lung metastasis of mice was observed after tail vein injection of OE-miR-15a-5p + 31-5p-OE-Mir100hg-LLC (red); **L**-**M** HE staining was used to observe and quantify lung metastases. (*p < 0.05, **p < 0.01, ***p < 0.001)

One of the mechanisms of lncRNA affecting tumor metastasis can be achieved by its activity as a "miRNA sponge" to inhibit the expression of miRNAs and thus affect the downstream regulatory network in the cytoplasm [28]. We first determined that Mir100hg was distributed in both the nucleus and cytoplasm in LLC and nucleoplasmic separation experiments (Fig. 4A-B). It is known that miRNAs can exist in the cytoplasm as miRNA ribonucleoprotein complexes and act as gene silencing through the Ago2 protein [29]. Therefore, to determine whether Mir100hg works via CeRNA (Competing endogenous RNAs), we performed RIP on LLC cells using antibodies to Ago2. RNA levels in immunoprecipitation were measured by qRT-PCR. Compared with control IgG immunoprecipitation, Mir100hg was preferentially enriched in ago2-containing miRNPs (Fig. 4C). These results suggest that Mir100hg may utilize a CeRNA regulatory mechanism through miRNA.

Next, we combined dry-lab bioinformatics analyses and wet-lab biological experiments to identify miRNAs that might be targeted by Mir100hg (Fig. 4D). The candidate miRNA dataset 1 was obtained by first predicting the miRNAs that are subjected to Mir100hg targeting through the Annoloc website. Mir100hg was highly expressed in LLC-SD, implying that genes regulated by Mir100hg through CeRNA may be highly expressed in LLC-SD. We then analyzed the RNA-seq results of LLC-SD and LLC cells and selected the genes that were up-regulated in LLC-SD (logFC > 1, *P* < 0.05) as candidate gene set. The candidate miRNA dataset 2 was obtained by TargetScan database analysis. Next, we intersected the two candidate miRNA datasets and obtained 37 miRNAs that were potentially regulated by Mir100hg (Fig. 4E). For biological validation, we measured the expression changes of these miRNAs upon overexpression or down-regulation of Mir100hg. We found that the expression of miR-15a-5p and miR-31-5p was negatively correlated with the expression of Mir100hg (Fig. 4F). Furthermore, we confirmed that Mir100hg could directly target miR-15a-5p and miR-31-5p by luciferase assay (Fig. 4G).

miR-15a-5p and miR-31-5p are two well-characterized tumor suppressor miRNAs that inhibit metastasis [30, 31]. Our hypothesis is that cytoplasmic Mir100hg augments the metastatic activity of lung cancer cells by targeting miR-15a-5p and miR-31-5p and acting through the CeRNA regulatory mechanism that leads to the relief of the tumor suppressive activity of miR-15a-5p and miR-31-5p. To test this hypothesis, we overexpressed miR-15a-5p and miR-31-5p individually or jointly (co-overexpression) in OE-Mir100hg-LLC (Fig. 4H). The effects of miR-15a-5p and miR-31-5p overexpression or co-overexpression on the pro-metastatic activity of Mir100hg were explored both in vivo and in vitro. Unexpectedly, overexpression of miR-15a-5p or miR-31-5p alone had no significant effect on the migration and invasion ability of LLC cells (Supplementary Fig. 1A). However, co-overexpression of miR-15a-5p and miR-31-5p significantly weakened the migration and invasion ability of OE-Mir100hg-LLC cells in vitro (Fig. 4I-J). To assess whether miR-15a-5p and miR-31-5p mediate the pro-metastatic activity of Mir100hg in vivo, rescue experiments were performed (Methods Animal experiments). From fluorescence photography and HE staining, it was evident that co-overexpression of miR-15a-5p and miR-31-5p in OE-Mir100hg-LLC successfully abolished the stimulating effect of Mir100hg overexpression on metastatic colonization of LLC in vivo (Fig. 4K-L).

### Mir100hg-miR-15a-5p/31-5p enhances lung cancer metastasis through glycolysis

**Fig. 5:**
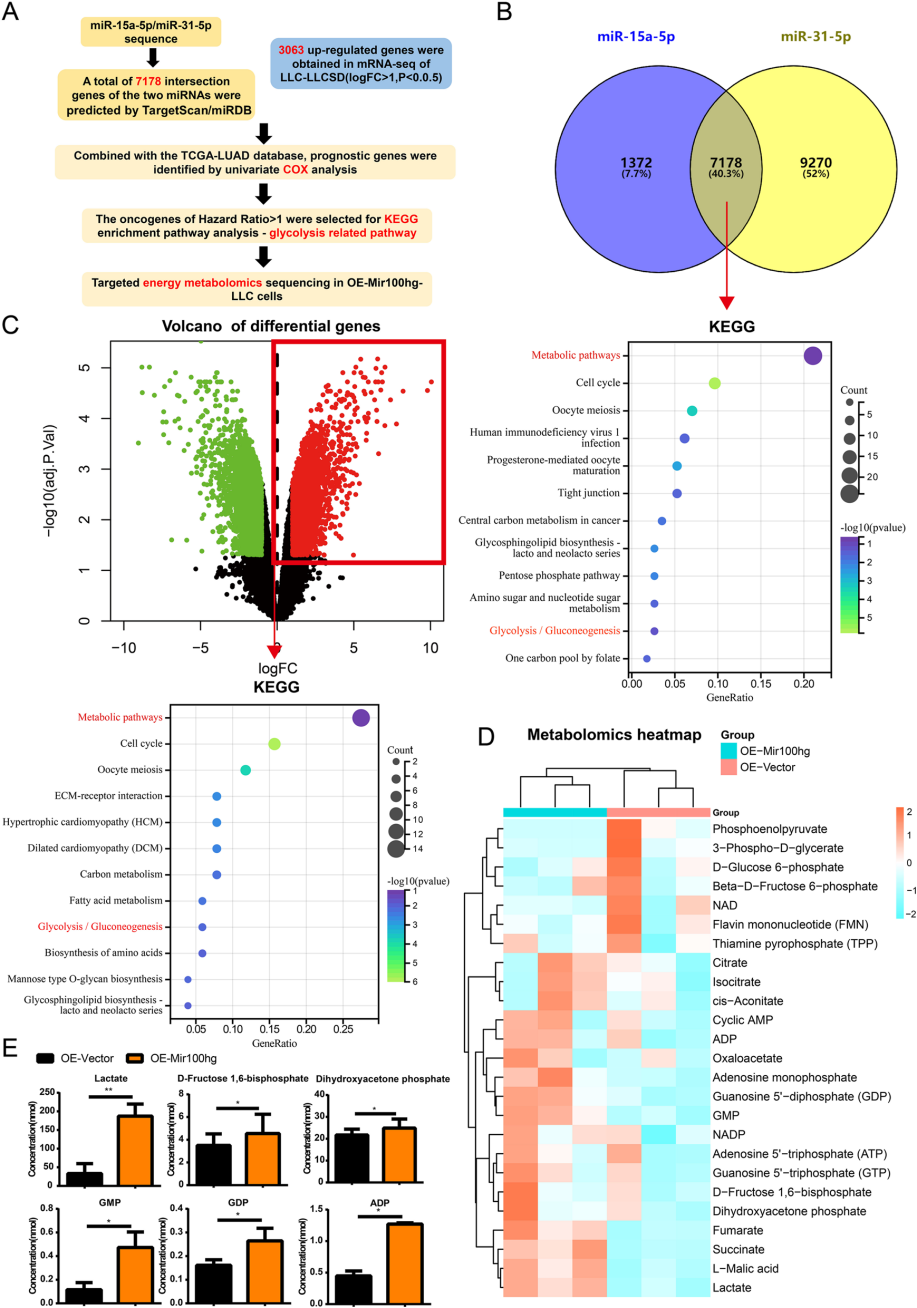
Mir100hg-miR-15a-5p/31-5p axis was enriched in the glycolytic pathway. **A** Schematic representation of inference to glycolytic pathway by miR-15a-5p and miR-31-5p sequences combined with LLC-SD and LLC sequencing data; **B** Intersection of the predicted target genes of miR-15a-5p and miR-31-5p and KEGG enrichment results of the poor prognostic genes screened by Cox regression; **C** The volcano map of differential genes between LLC-SD and LLC and the KEGG enrichment results of the poor prognostic genes screened by Cox regression; **D** Heatmap of targeted energy metabolomics of OE-Mir100hg-LLC **E** Bar graph of differential glycolysis-related metabolites of OE-Mir100hg-LLC

In order to find the potential mechanism by which LLC-SD Exosomal Mir100hg promotes LLC metastatic activity through miR-15a-5p/miR-31-5p, we performed bioinformatics analysis combined with RNA sequencing and targeted metabolomics sequencing to predict significant enrichment of biological processes as follows (Fig. 5A): First, we predicted 7178 target genes jointly regulated by miR-15a-5p and miR-31-5p through miRDB and TargetScan websites. Meanwhile, 3063 up-regulated genes were selected from the RNA-seq results of LLC-SD and LLC cells. Then, we combined the two gene sets and conducted survival analysis using the TCGA-LUAD database, followed by COX regression analysis to obtain the genes indicating poor prognosis with Hazard Ratio greater than 1. KEGG was used for pathway enrichment (Fig. 5B-C). Glycolysis/Gluconeogenesis showed significant enrichment among metabolic pathways. This implies that Mir100hg-miR-15a-5p/miR-31-5p may enhance the metastatic ability of LLC cells through glycolysis.

Glucose is the most important energy supply for tumor development. Aerobic glycolysis, also known as the Warburg effect, is of particular importance. We hypothesized that Mir100hg may increase glycolysis through inhibition of miR-15a-5p/miR-31-5p, thereby enhancing the metastatic activity of lung cancer cells. Therefore, targeted energy metabolite sequencing was used to explore the metabolite changes in OE-Mir100hg-LLC cells (Fig. 5D). We found that lactate, the major metabolite produced during glycolysis, was aberrantly increased in Mir100hg overexpression group. Some intermediate products of glycolysis, including D-Fructose 1,6-bisphosphate, Dihydroxyacetone phosphate were increased in OE-Mir100hg-LLC cells (Fig. 5E). These results indicate that Mir100hg stimulated glycolytic activity and caused concomitant changes in glycolysis-related metabolites.

**Fig. 6:**
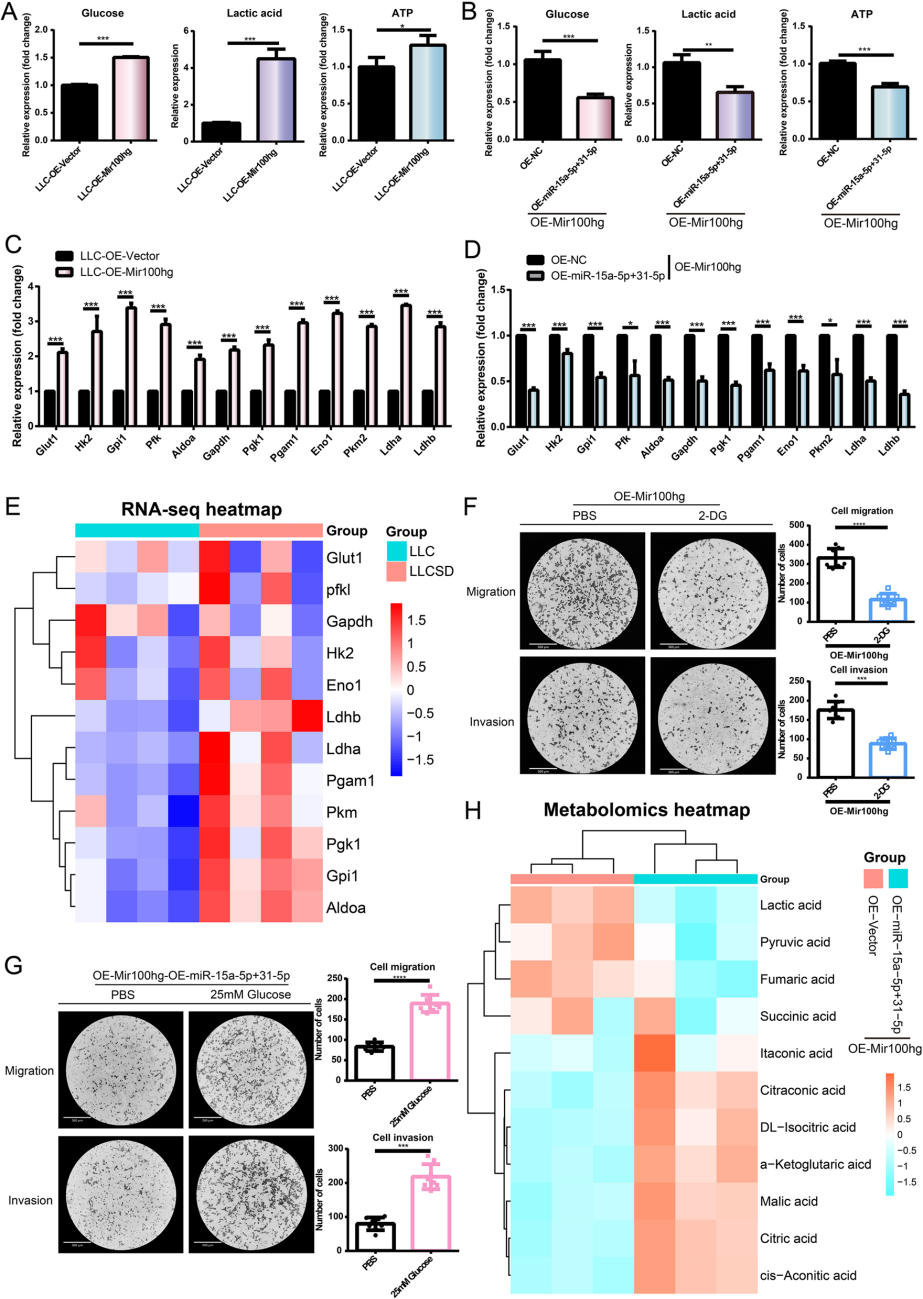
Mir100hg-miR-15a-5p/31-5p enhanced lung cancer metastasis through glycolysis. Levels of glucose, lactate and ATP in OE-Mir-100hg-LLC cells **A** and in OE-miR-15a-5p + 31-5p-OE-Mir-100hg-LLC cells **B**. mRNA levels of key rate-limiting enzymes of glycolysis in OE-Mir-100hg-LLC cells **C** and in OE-miR-15a-5p + 31-5p-OE-Mir-100hg-LLC cells; **E** Heat map of differential expression of key glycolytic enzymes in RNA-seq data of LLC-SD and LLC; **F** The migration and invasion of OE-Mir100hg-LLC treated with 1mM 2-DG for 20h; **G** migration and invasion of OE-miR-15a-5p + 31-5p-OE-Mir100hg-LLC treated with 25mM glucose for 20h; (H) Differential metabolite heatmap related to glycolysis and TCA in the untargeted metabolomics of OE-miR-15a-5p + 31-5p-OE-Mir100hg-LLC. (*p < 0.05, **p < 0.01, ***p < 0.001)

Next, we examined the expression changes of glucose, lactate, and ATP in OE-Mir100hg-LLC cells and OE-miR-15a-5p/31-5p-OE-Mir100hg-LLC cells. We found that overexpression of Mir100hg increased the intracellular content of all three metabolites (Fig. 6A). In contrast, the expression of the three metabolites were decreased after co-overexpression of both miR-15a-5p and miR-31-5p in OE-Mir100hg-LLC cells. This means that overexpression of miR-15a-5p and miR-31-5p could inhibit the enhanced glycolytic activity caused by Mir100hg overexpression. (Fig. 6B). The expression of some key enzymes in the process of glycolysis was also measured. We observed upregulation of Glut1/ Gapdh/Hk2/Pgk1/Gpi/Pgam1/Pfkl/Eno1/Aldoa/Pkm2/Ldha/Ldhb mRNA expression by Mir100hg (Fig. 6C). In contrast, co-overexpression of miR-15a-5p and miR-31-5p resulted in reduced expression levels of these rate-limiting enzymes (Fig. 6D). Differential expression of these rate-limiting enzymes was detected in the mRNA sequencing data of LLC-SD and LLC. Several rate-limiting enzymes exhibited higher expression in LLC-SD in which Mir100hg expression was higher than that of LLC (Fig. 6E). To confirm that Mir100hg-miR-15a-5p/miR-31-5p affected lung cancer cell metastasis by enhancing glycolysis, functional recovery experiments of glycolysis were performed. The enhanced migration and invasion of LLC induced by Mir100hg overexpression was reversed by adding glycolytic inhibitor 2-DG to OE-Mir100hg-LLC cells (Fig. 6F). In contrast, adding glucose to stimulate glycolysis in OE-miR-15a-5p + 31-5p-OE-Mir100hg-LLC cells reversed the reduced migration and invasion ability (Fig. 6G). To further determine the overall effect of miR-15a-5p/miR-31-5p on energy metabolic activity in tumor cells, we used untargeted metabolomics to examine the effects of miR-15a-5p/miR-31-5p on cellular metabolism. (Fig. 6H). The results showed that the levels of lactate and pyruvate, the two major products in the glycolytic process, were significantly decreased in OE-miR-15a-5p + 31-5p-OE-Mir100hg-LLC compared with that of the control group, confirming that miR-15a-5p/31-5p inhibited the glycolytic.

**Fig. 7:**
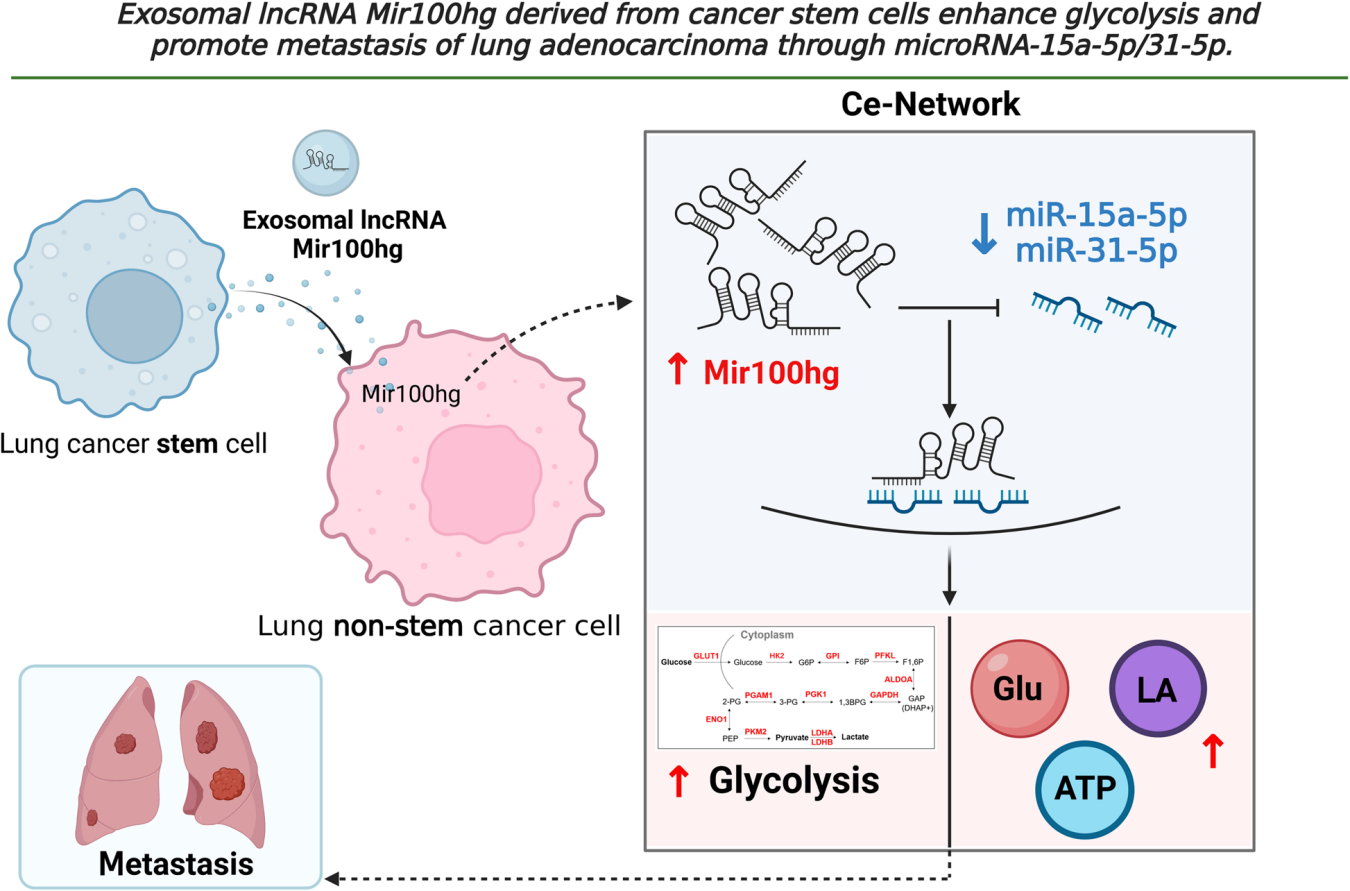
Schematic presentation of the mechanism- “Exosomal lncRNA Mir100hg derived from cancer stem cells enhances glycolysis and promote metastasis of lung adenocarcinoma through mircroRNA-15a-5p/31-5p”

In summary (Fig. 7), we have revealed for the first time that the highly expressed lncRNA Mir100hg in the lung cancer stem cell LLC-SD can be delivered to targeted non-stemness lung cancer cells LLC by exosomes. Upon entering LLC, Mir100hg targeting of miR-15a-5p and miR-31-5p leads to the relief of the inhibitory effect of both miRNAs on glycolysis, which in turn enhances the metastatic activity of LLC. Thus, through the Exosomal Mir100hg/miR-15a-5p/31-5p/glycolytic axis, Lung cancer stem cells can transfer its "metastatic capacity" to low-metastatic non-stemness lung cancer cells.

## Discussion

MIR100HG was firstly reported to be involved in the survival fate of human MSCS (Mesenchymal Stem cell) [32]. Expressed at high levels in various tumors, MIR100HG enhances oncogenesis as well as tumor progression [33–35]. Despite the investigation of mechanisms underline the pro-oncogenic activity of MIR100HG in various tumors, its role in lung cancer metastasis and in mediating the communication between two heterogeneous cell populations in the metastatic microenvironment of lung has not been investigated prior to the present study.

In this study, we have investigated the interaction between lung cancer stem cells with high metastatic capability (LLC-SD) and non-stemness cancer cells with low metastatic potential (LLC). Our findings reveal firstly that lncRNA Mir100hg, highly expressed in lung cancer stem cells, can be transferred from cancer stem cells to non-stemness cancer cells through exosomes. Moreover, Mir100hg directly targets miR-15a-5p/miR-31-5p to enhance the glycolysis of non-stemness cancer cells, thereby enhancing their metastatic ability. Our study uncovers the complexity of the mechanisms by which lncRNA Mir100hg affects lung cancer metastasis and have enriches our understanding of the molecular and physiological mechanisms by which exosomal non-coding lncRNAs regulate tumor metastasis.

In our previous study, we discovered that secretory proteins in the paracrine pathway can mediate the communications between tumor cells with different metastatic ability [36]. Exosomes, a new category of paracrine substances, have more complex contents and higher delivery efficiency [37]. Exosomal lncRNAs have been shown to play a crucial role in the initiation and development of tumors and serve as mediators of communication between tumor cells and other types of cells in the tumor microenvironment [38–40]. For instance, lncRNA LNMAT2 can be imported into bladder cancer exosomes through hnRNPA2B1 and internalized by human lymphatic endothelial cells, ultimately leading to lymphangiogenesis and lymphatic metastasis [41]. Additionally, lncRNA LINC00623 can be encapsulated by exosomes and delivered to promote the tumorigenicity and migration of pancreatic ductal carcinoma in vitro and in vivo [42]. These studies show the potential for exosomal lncRNAs to be used as diagnostic biomarkers for lung cancer. The control of exosome production and the levels of pro-oncogenic lncRNA may offer a new way to treat lung cancer. Nevertheless, whether lung cancer stem cells can deliver lncRNAs via exosomes to enhance the invasiveness of extravasated non-stemness tumor cells during tumor metastasis remains unclear. Our study demonstrates for the first time that lung cancer stem cells can deliver Mir100hg through exosomes to enhance the metastatic ability of non-stemness lung cancer cells. This mechanism highlights the complexity of lung cancer metastasis and enriches our understanding of the molecular and physiological mechanisms of exosomal lncRNA in regulating tumor metastasis by demonstrating the transmission of LncRNA Mir100hg by exosomes to regulate the interaction between two heterogeneous cells in the tumor microenvironment.

Intriguingly, our research group conducted RNA-PULLDOWN assays with Mir100hg (data not shown) and identified a cohort of candidate genes by utilizing mass spectrometry that may directly bind to and act with Mir100hg. Among these candidates, hnRNPA2B1, has been reported to be involved in the sorting of exosomes in order to package lncRNAs. Lu et’ al. reported that MIR100HG interacts with hnRNPA2B1 to stabalize TCF7L2 mRNA in an m6A-dependent manner. However, the mechanism by which MIR100HG directly binds to hnRNPA2B1 to impact the entrapment and transportation of MIR100HG in exosomes was not examined [43]. Our forthcoming research will focus on how hnRNPA2B1 regulates the specific entry of Mir100hg into the exosomes, ascertaining whether exosomal ingestion of lncRNA is spatiotemporally regulated or it is a selective biological event mediated by hnRNPA2B1. This will address the key question of how exosomal lncRNA is generated and transported into exosomes.

Metabolic reprogramming is a significant feature of cancer [44]. The energy supply of normal cells is mainly through oxidative phosphorylation, while cancer cells rely on aerobic glycolysis accompanied by Warburg effect to obtain stronger proliferation ability [45]. However, how tumor metabolism is regulated by lncRNAs remains largely unknown. Xu et al. demonstrated that lncRNA AGPG could remodel the aerobic glycolysis pathway of esophageal cancer by regulating the post-translational modification of metabolic enzyme PFKFB3, thereby promoting the proliferation of esophageal cancer [46]. Song's team revealed for the first time that tumor-associated macrophages (TMS) in the tumor microenvironment regulate glucose metabolism by secreting exosomes-encapsulated lncRNA HISLA [47]. These studies indicate that LncRNA can regulate tumor glycolysis and affect the malignant phenotype of tumors through autocrine effect on its own or exosome delivery into the receipient cells. The results of the present study have revealed that lncRNA Mir100hg can be transferred from lung CSCs to the non-stemness cancer cells via exosomes and enhances the glycolytic activity of non-stemness tumor cells, leading to the accumulation of lactate.

The abnormal lactate accumulation induced by exosomal Mir100hg also aroused our interest in lactate. In 2019, Zhao's team proposed the concept of lactylation modification, which is a new type of protein post-translational modification involving lactic acid [48]. At present, there is no relevant report on lncRNA involved in lactate modification, which will be one of our future research directions. We believe that elucidating the mechanism of tumor metabolic remodeling and expanding the diagnosis and treatment plan based on tumor metabolic abnormalities will have important clinical significance for the precise diagnosis and treatment of cancer.

In most studies of CeRNA mechanism, researchers usually study the lncRNA-miRNA-mRNA axis, highlighting the "one-to-one" precise regulation between miRNA and mRNA. However, the complexity of cancer predestined a variety of underline regulatory mechanisms. In this study, taking Mir100hg as the starting point, we predict and verify the target miRNAs and downstream biological processes that may be involved in Mir100hg action by bioinformatics analysis and modeling and combined with biological experimentation. We have revealed a complex and "one-to-many" regulatory network. Interestingly, our results show that OE-miR-15a-5p or OE-miR-31-5p alone does not significantly inhibit the Mir100hg-enhanced metastatic ability of lung cancer cells, and only the co-targeting of these two miRNAs has the effect on altering the metastatic ability of non-stemness cells both in vivo and in vitro. This suggests that the "one-to-one" CeRNA mechanism may not always capture the intricacies of tumor metastasis. We believe that this "one-to-many" regulatory mechanism that we have delineated can help us to take a more holistic view of tumor metastasis. From lncRNA to miRNA to glycolysis, a complex and vast regulatory network behind tumor metastasis has been revealed. We expect that in the future, with the improvement of high-throughput sequencing and experimental technology, more studies will reveal the corresponding theoretical and molecular mechanisms of tumor heterogeneity, and further promote the new understanding of its formation and tumor evolution.

## Conclusions

In summary, we have found for the first time that lncRNA Mir100hg is highly expressed in lung cancer stem cells, which can be encapsulated into exosomes and transferred to non-stemness tumor cells, and enhance glycolysis and metastasis of lung cancer cells through miR-15a-5p/31-5p. Although challenging, the development of therapies targeting exosomal lncRNA Mir100hg may be beneficial in metastatic lung cancer. With further research in the future, Mir100hg may become a new diagnostic marker and clinical therapeutic target for cancer.

### Supplementary Information

**Additional file 1.** 

**Additional file 2.**

## Data Availability

The datasets used and analysed during the current study are available from the corresponding author on reasonable request.

## Abbreviations

CSCs: Cancer stem cells
EVs: Extracellular vesicles
FBS: Fetal bovine serum
FISH: Fluorescence in situ hybridization
LLC: Lewis lung cancer cells
LLC-SD: Lung cancer stem cells
GEO: Gene Expression Omnibus
CeRNA: Competing endogenous RNAs
RIP: RNA-binding protein immunoprecipitation
KEGG: Kyoto Encyclopedia of Genes and Genomes
