# Supplementary material for: Exosomal lncRNA Mir100hg derived from cancer stem cells enhance glycolysis and promote metastasis of lung adenocarcinoma through mircroRNA-15a-5p/31-5p
Source: Cell Commun Signal. 2023 Sep 21;21:248. doi: 10.1186/s12964-023-01281-3 (PMC10512609; doi:10.1186/s12964-023-01281-3)
Supplement: Supplementary file 2 — Additional file 1. [file 12964_2023_1281_MOESM1_ESM.docx]

**Supplemental Information:**

# Exosomal lncRNA Mir100hg derived from cancer stem cells enhance glycolysis and promote metastasis of lung adenocarcinoma through mircroRNA-15a-5p/31-5p.

Lei Shi^1^#, Bowen Li^1^#, Yuhan Zhang^1^，Yu ting Chen^1^, Jiyu Tan^1^, Yan Chen^1^, Jie Li^2^, Meng Xiang^1^, H. Rosie Xing^1^*, Jianyu Wang^2^*.

^1^State Key Laboratory of Ultrasound in Medicine and Engineering, College of Biomedical Engineering, Chongqing Medical University, Chongqing, 400016, Chin

^2^Institute of Life Sciences, Chongqing Medical University, Chongqing, 400016, China

***Corresponding**

Jianyu Wang: [102758@cqmu.edu.cn](mailto:102758@cqmu.edu.cn)

H. Rosie Xing: [102643@cqmu.edu.cn](mailto:102643@cqmu.edu.cn)

Tel.: +86-023-63662443 (J.W.), +86-023-63738563 (H.R.X.);

Fax.: +86-023-63662443 (J.W.), +86-023-68486646 (H.R.X.).

Present address: Yi Xue Yuan Road, Yuzhong District Chongqing, P.R. China 400016.

#### Supplementary Tables:

**Table1:** The primers and siRNA sequences:

| GAPDH | Forward: TGATGGGTGTGAACCACGAG |
| --- | --- |
|  | Reverse: AGTGATGGCATGGACTGTGG |
| TBP | Forward: AAGAGAGCCACGGACAACTG |
|  | Reverse: TTCACATCACAGCTCCCCAC |
| β-actin | Forward: CTACCTCATGAAGATCCTGACC |
|  | Reverse: CACAGCTTCTCTTTGATGTCAC |
| U6 | Forward: CTCGCTTCGGCAGCACA |
|  | Reverse: AACGCTTCACGAATTTGCGT |
| Mir100hg-1 | Forward: CCAGTGACCAGCTGAAGAGG |
|  | Reverse: GTGTTCTGGGCTTCTCTGCT |
| Mir100hg-2 | Forward: GGCAGAGGAGATCCACATGG |
|  | Reverse: GGGAGGGGAGATACCTGTGT |
| Rab27a | Forward: TAGCCGAGGGGTCATGAAGA |
|  | Reverse: ACAAAGGGAGTTTTGGGCCA |
| miR-15a-5p | Forward: ACGGAACTAGCAGCACATAATG |
|  | Reverse: CAGTGCAGGGTCCGAGGT |
| miR-31-5p | Forward: AACGGCAGGCAAGATGCTGG |
|  | Reverse: ATCCAGTGCAGGGTCCGAGG |
| Gpi | Forward: CAGAGACAGCAAAGGAGTGG |
|  | Reverse: GTAGACAGGGCGACAAAGTG |
| Eno1 | Forward: CGCCATGTCTATTCTCAGGATC |
|  | Reverse: AGTTCTAGGGCCTCGTAGATG |
| Pfkl | Forward: GGAAAGCCTATCTCATCCAGC |
|  | Reverse: CCATACCCATCTTGCTACTCAG |
| Aldoa | Forward: CCCCAAGTTATCAAGTCCAAGG |
|  | Reverse: GTTCAGACAGCCCATCCAG |
| Pgam1 | Forward: GAAAAGGGTCTTGATTGCCG |
|  | Reverse: GTTCATAGACGATAGGGATGCC |
| Glut1 | Forward: GATTGGTTCCTTCTCTGTCGG |
|  | Reverse: CCCAGGATCAGCATCTCAAAG |
| Hk2 | Forward: TCAAAGAGAACAAGGGCGAG |
|  | Reverse: AGGAAGCGGACATCACAATC |
| Pgk1 | Forward: AACCTCCGCTTTCATGTAGAG |
|  | Reverse: GACATCTCCTAGTTTGGACAGTG |
| Pkm | Forward: CCATTCTCTACCGTCCTGTTG |
|  | Reverse: TCCATGTAAGCGTTGTCCAG |
| Ldha | Forward: GCTCCCCAGAACAAGATTACAG |
|  | Reverse: TCGCCCTTGAGTTTGTCTTC |
| Ldhb | Forward: ACAAGTGGGTATGGCATGTG |
|  | Reverse: ACAATTTTCGGAGTCTGGAGG |

**Table 2:** Antibodies

| Rab27a | Cat No. 17817-1-AP | 1:2000 | Proteintech |
| --- | --- | --- | --- |
| CD63 | Cat No. 25682-1-AP | 1:1000 | Proteintech |
| CD81 | Cat No. 66866-1-Ig | 1:500 | Proteintech |
| Alix | Cat No. 12422-1-AP | 1:1500 | Proteintech |
| Albumin | Cat No. 66051-1-Ig | 1:1500 | Proteintech |
| GAPDH | Cat No. 60004-1-Ig | 1:5000 | Proteintech |
| AGO2 | Cat No. 67934-1-Ig | 1:1000 | Proteintech |

**Table 3:** sequence of plasmid:

pGL3-Basic：
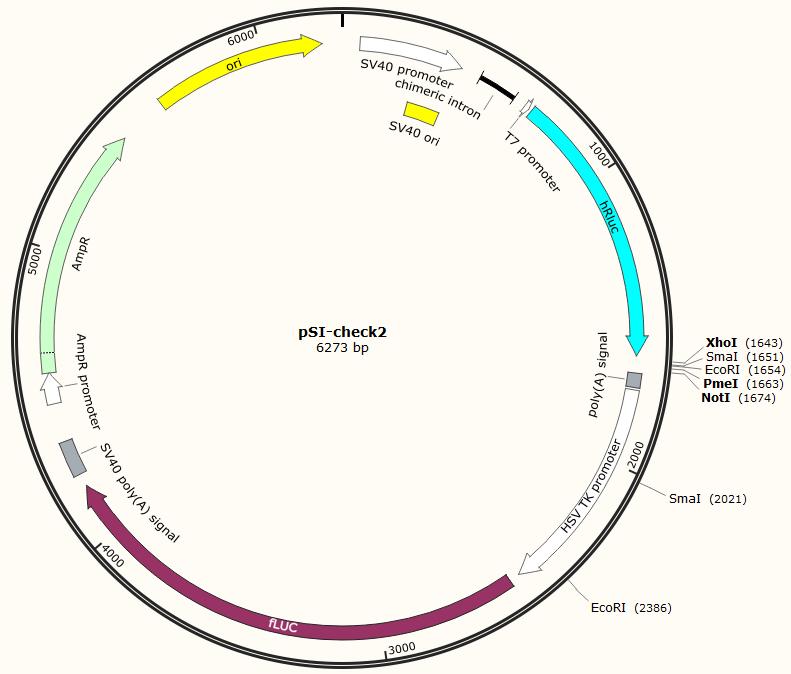


sequence of plasmid:

| **Mir100hg- Wt** | AAGGTGAAGGGCCTCCACTTCAGCCAGGAGGACGCTCCAGATGAAATGGGTAAGTACATCAAGAGCTTCGTGGAGCGCGTGCTGAAGAACGAGCAGTAATTCTAGGCGATCGCTCGAGCTGCTCCCGATGGCGCTCCTGGAAGCTCTGCAACTATGCTCCACTTCAAATGGGGAGGGTAGACCTGAAGCTTTCTTCTTTGTACACACTGACACAAGACTGCTGCTGCATTGATTCTTGAGCAAGGCGAACAATGGAAGAAAGCCACTTTTTGTTGTTAAAGTGAGGTAGCATTTCTGCTGTAGTTAGAAAGAGCATTGTCTCTCCAGTGTCCCAAATCACTTCCATTAAACAGTCAATGTGATAACTGTCTGTGACATGTTCTATTATCTTTCTAGTTGTTCTGATGTTCCTTTCAGCTGGTCCTATAGCCCCCTTTCTCAGTAATGTCTCCCCAGTGAAAGGAAAAAGACTGCAGAGGCTTCAGACTGACGGACGCTCATGCCAACTGTGAAGTTGAAACAAAGCCAGTGACCAGCTGAAGAGGCCCCTCCTGCCCAGCAGCCACCCACATGGTGCAGTTGCAGCCAGTAGTAATCTTTGCAAGAAGCAGAGAAGCCCAGAACACTCATGAGTCAAAACCATGCCCTTCCTTGTGTGTATCCCTGTCTCGTTCAGATTGATGCTCCTTCCATTTGGATCCTGGTATGAATATGCTTGTTACTGTACGAGTGTAAAAAGCTTTGCCCAAATGTGTTAATGTATGAAGAGACTGGATTTTTTTTGGGGGGGGGAGAGAGTTATTTTTATCAAAACACAGTGTTCGTTCTTGAGAGTGATCTAGAAAATAAAAAGCAGTTGGAAAAGACAGCAAAGCAGTCACAGGCGATCCACAGAGATGCTCACACCACCACACAGAAACACCACACCATGCTCCCAGACTCTGAGCAGGGAGCTTGACTCTGTGACATACTATGGCTGCTGCCAGGGGTCAACGTCAAGCCAGACAAAGGTGCAGAAGCTTCCTGAAGTGTAGTGATAAACTGAGTCAGGAGACAATAAGAACAGCAACGACACAAAAGACAACGTCCTTGGAGGGAGCCAAGTGGGTTTGCTTAAGGAAGGATGCTCCCTTAAAGAGAGAATGGGAAATCAACCTTTAAAGGATGTAAAAACAAAACTAGATAAATGGGAAGATAGGAGAAAATAGAGTCCTTAATGCAAGCATAAGGGTAAGCAGCAGACGGGCAGAGGAGATCCACATGGTGAGTCTTGGAGACTACACATATCTCGTCTTGATTTTGCCTGACACTTGGAACCGCTGTCTGTGTGTCTAGTAGAGATGCTGCACACAGGTATCTCCCCTCCCCCACCCCGAATGTCACCTAAACCAAACTAGAACTAAAGTTCCAGTTCTGTGTCACTACAAAGATTGGTTGGTGATTCCTGGCGGCCGCTGGCCGCAATAAAATATCTTTATTTTCATTACATCTGTGTGTTGGTTTTTTGTGTGAGGATCTAAATGAGTCTTCGGACCTCGCGGGGGCCGCTTAAGCGGTGGTTAGGGTTTGTCTGACGCGGGGGGAGGGG |
| --- | --- |
| **Mir100hg-mut** | GGGGGCTCCTTCGGAGGGAGGAGCTCCAGATGAATGGGTAAGTACATCAAGAGCTTCGTGGAGCGCGTGCTGAAGAACGAGCAGTAATTCTAGGCGATCGCTCGAGCTGCTCCCGATGGCGCTCCTGGAAGCTCTGCAACTATGCTCCACTTCAAATGGGGAGGGTAGACCTGAAGCTTTCTTCTTTGTACACACTGACACAAGACaGgTcCaGgATTGATTCTTGAGCAAGGCGAACAATGGAAGAAAGCCACTTTTTGTTGTTAAAGTGAGGTAGCATTTCTGCTGTAGTTAGAAAGAGCATTGTCTCTCCAGTGTCCCAAATCACTTCCATTAAACAGTCtAaGaGtTAACTGTCTGTGACATGTTCTATTATCTTTCTAGTTGTTCTGATGTTCCTTTCAGCTGGTCCTATAGCCCCCTTTCTCAGTAATGTCTCCCCAGTGAAAGGAAAAAGACTGCAGAGGCTTCAGACTGACGGACGCTCATGCCAACTGTGAAGTTGAAACAAAGCCAGTGACCAGCTGAAGAGGCCCCTCCTGCCCAGCAGCCACCCACATGGTGCAGTTGCAGCCAGTAGTAATCTTTGCAAGAAGCAGAGAAGCCCAGAACACTCATGAGTCAAAACCATGCCCTTCCTTGTGTGTATCCCTGTCTCGTTCAGATTGATGCTCCTTCCATTTGGATCCTGGTATGAATATGCTTGTTACTGTACGAGTGTAAAAAGCTTTGCCCAtAaGaGTTAATGTATGAAGAGACTGGATTTTTTTTGGGGGGGGGAGAGAGTTATTTTTATCAAAACACAGTGTTCGTTCTTGAGAGTGATCTAGAAAATAAAAAGCAGTTGGAAAAGACAGCAAAGCAGTCACAGGCGATCCACAGAGATGCTCACACCACCACACAGAAACACCACACCATGCTCCCAGACTCTGAGCAGGGAGCTTGACTCTGTGACATACTATGcCaGgTcCCAGGGGTCAACGTCAAGCCAGACAAAGGTGCAGAAGCTTCCTGAAGTGTAGTGATAAACTGAGTCAGGAGACAATAAGAACAGCAACGACACAAAAGACAACGTCCTTGGAGGGAGCCAAGTGGGTTTGCTTAAGGAAGGATGCTCCCTTAAAGAGAGAATGGGAAATCAACCTTTAAAGGATGTAAAAACAAAACTAGATAAATGGGAAGATAGGAGAAAATAGAGTCCTTAATGCAAGCATAAGGGTAAGCAGCAGACGGGCAGAGGAGATCCACATGGTGAGTCTTGGAGACTACACATATCTCGTCTTGATTTTGCCTGACACTTGGAACCGCTGTCTGTGTGTCTAGTAGAGAaGgTcCACACAGGTATCTCCCCTCCCCCACCCCGAATGTCACCTAAACCAAACTAGAACTAAAGTTCCAGTTCTGTGTCACTACAAAGATTGGTTGGTGATTCCTGAATAGAAGTCATGATTCTTCTGTTCGTGATTGCTTGTTACCAAGTTTATTCCATACAGAAAATGTTTTGCATATTAAATCTCATGGTTGCTATGAAAACGACACATTATCCACCTTTCACAGGTGAAGGAACTGATTTTGGTGAGGTTGAGCGCCTGGCCAAGGTGCTACGTGCAGCTATGCACATTCAAGGTTGTGGTCATGAAACTCAGTCCCTAGTCTGACTTTTGTGATGAGTCCCAACACAGTTGTCACCTTTACCATAGCCCTTAATGCCTGTCTACAGAAAGACATGGCTTTTCTGCTGACTTACAGCCACAGTATTTCCTACTTCCATGCTGAATTATACCAGAATGATAAAACGTATTTTCATGGAAGAAAATTGAGAATTTAGTGAAATACACTGgTaGgCAGCAGTTTGTATGTTTCTTCTTCATTTTGGTCTGAGACTTTTGATTTAGTTCTGCATGAAATCTCAAAGTTTTAAGAGCAGTAAGGGAAGGGCCTTAAGTGCGGCCGCTGGCCGCAATAAAATATCTTTATTTTCATTACATCTGTGTGTGGTTTTTTTGGAGA |

#### Supplementary figures and legends:

Supplementary FIG. 1:


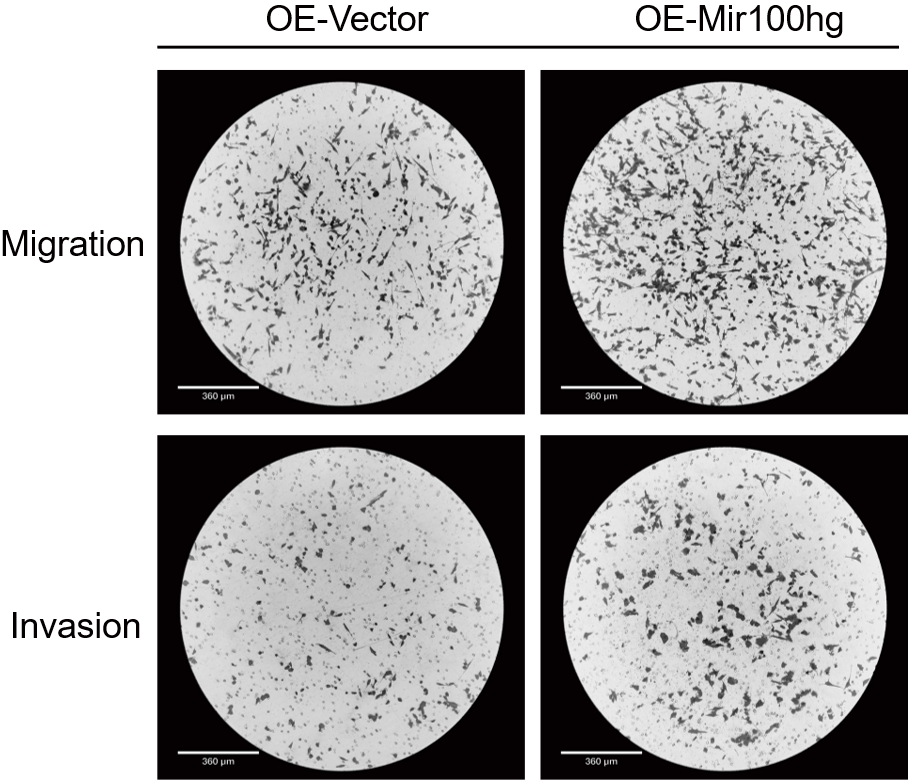


Transwell migration and invasion of OE-Vector-LLC and OE-Mir100hg-LLC.

Supplementary FIG. 2:


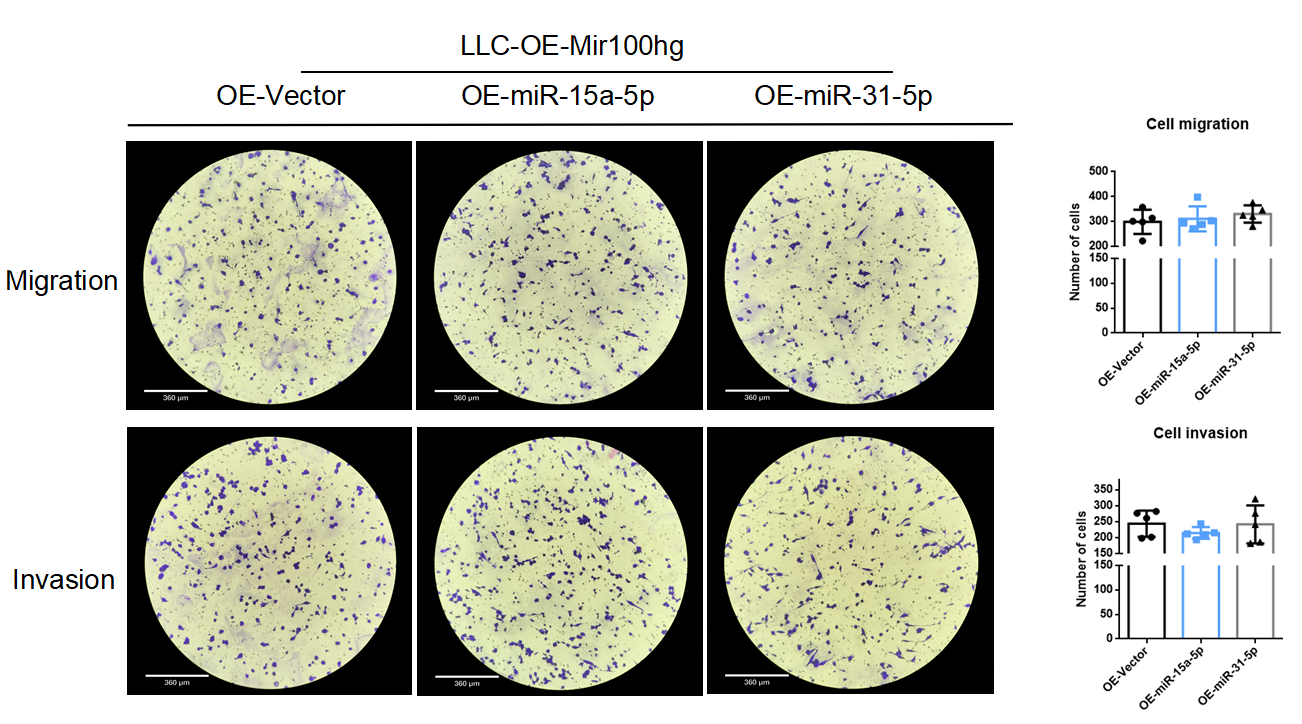


Transwell migration and invasion experiment and statistical diagram of miR-15a-5p and miR-31-5p separately overexpressed in OE-Mir100hg-LLC cells.

Supplementary FIG. 3:


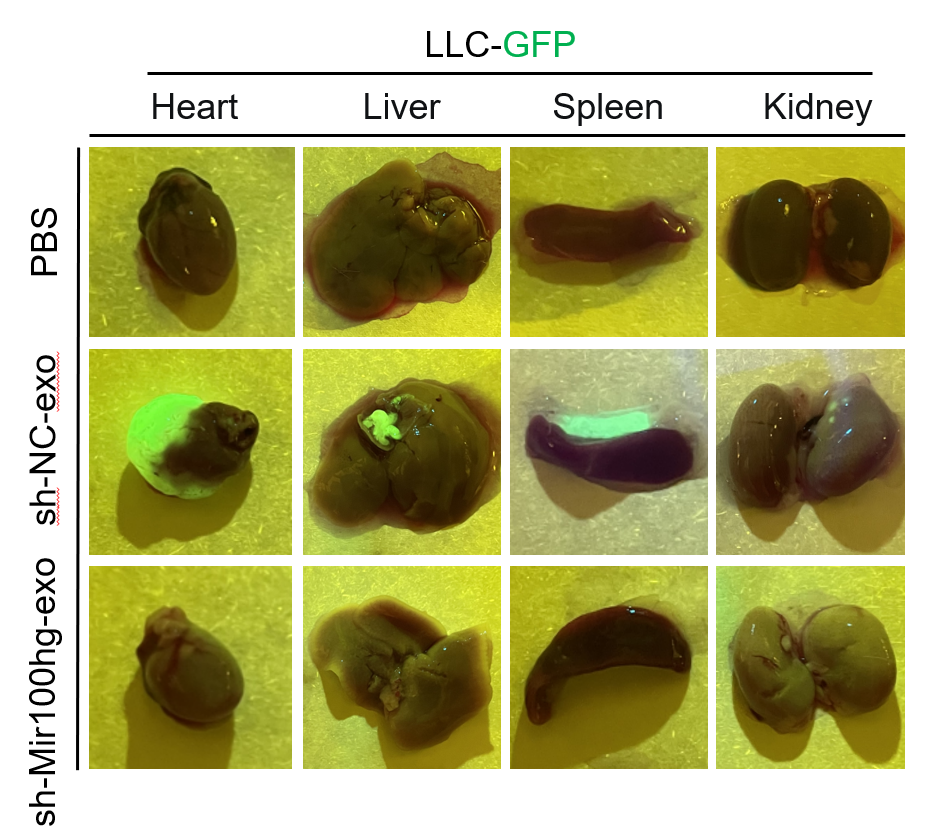


Metastasis of heart, liver, spleen and kidney tumors in different groups. (Green)
