# Supplementary figures and images for: Exosomal lncRNA Mir100hg derived from cancer stem cells enhance glycolysis and promote metastasis of lung adenocarcinoma through mircroRNA-15a-5p/31-5p
Source: Cell Commun Signal. 2023 Sep 21;21:248. doi: 10.1186/s12964-023-01281-3 (PMC10512609; doi:10.1186/s12964-023-01281-3)

**Uncropped Western blot images**


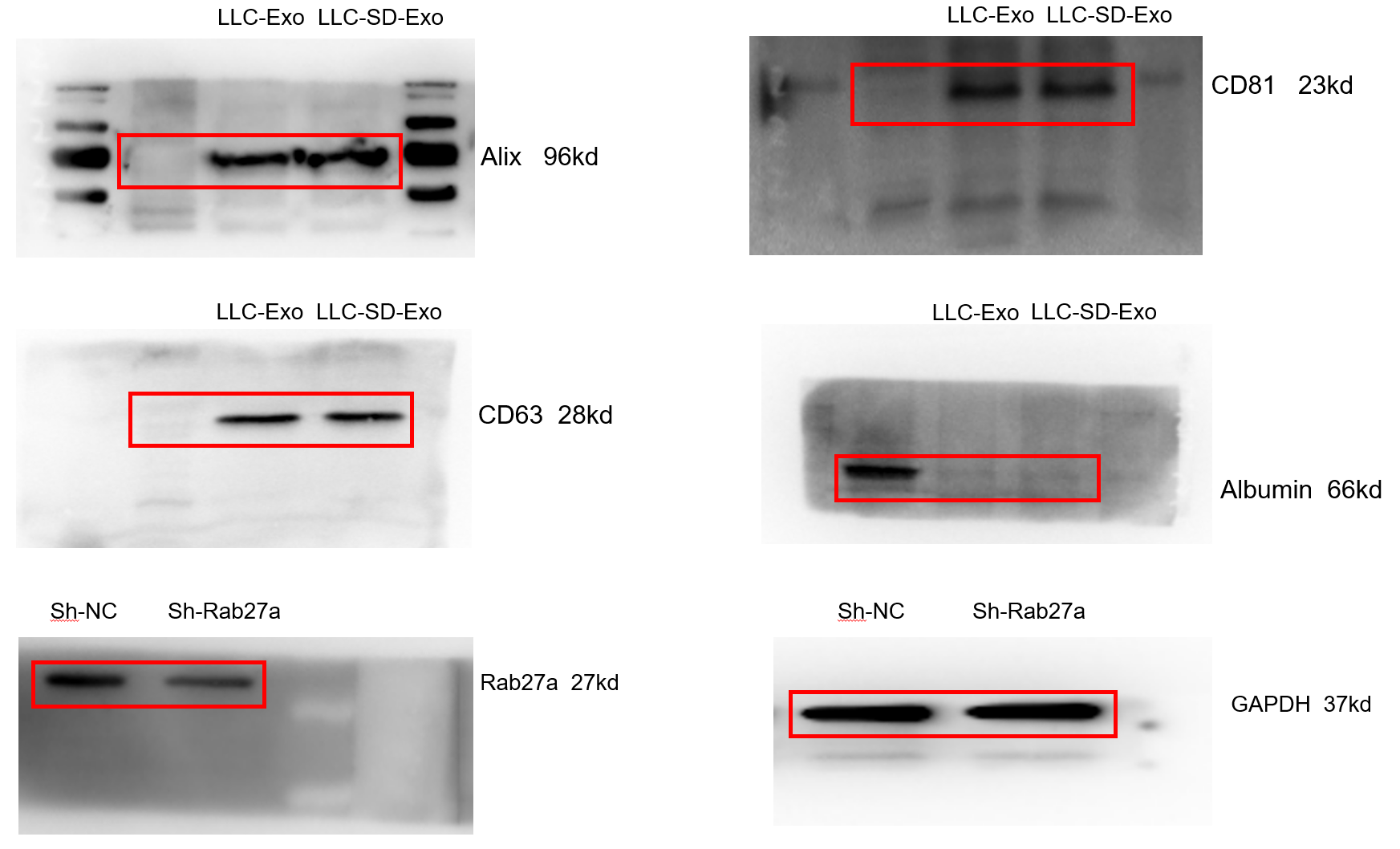

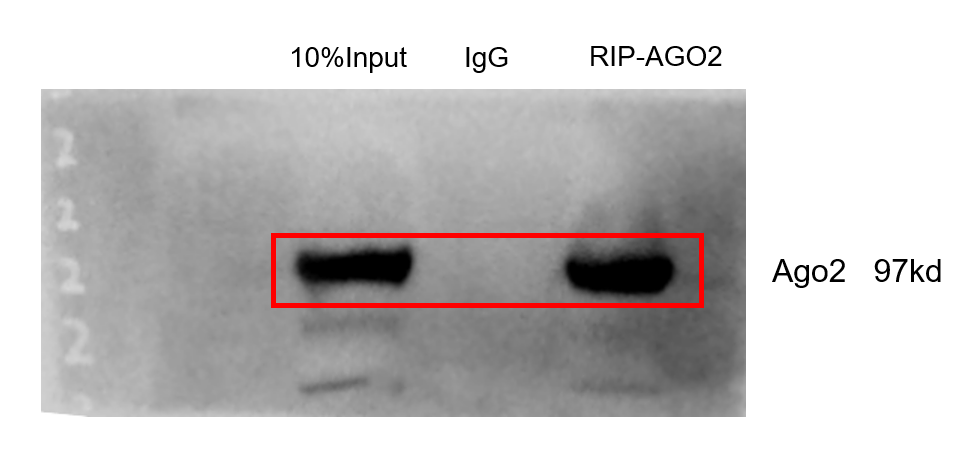

Supplement: Supplementary file 3 — Additional file 2. [file 12964_2023_1281_MOESM2_ESM.docx]
